# Colorectal Advanced Neoplasms Occur through Dual Carcinogenesis Pathways in Individuals with Coexisting Serrated Polyps

**DOI:** 10.1371/journal.pone.0098059

**Published:** 2014-05-21

**Authors:** Atsushi Yamada, Sachiko Minamiguchi, Yoshiharu Sakai, Takahiro Horimatsu, Manabu Muto, Tsutomu Chiba, C. Richard Boland, Ajay Goel

**Affiliations:** 1 Gastrointestinal Cancer Research Laboratory, Baylor Research Institute, Baylor University Medical Center, Dallas, Texas, United States of America; 2 Department of Gastroenterology and Hepatology, Graduate School of Medicine, Kyoto University, Kyoto, Japan; 3 Department of Diagnostic Pathology, Kyoto University Hospital, Kyoto, Japan; 4 Department of Surgery, Graduate School of Medicine, Kyoto University, Kyoto, Japan; 5 Department of Experimental Therapeutics, Institute for Advancement of Clinical and Translational Science (iACT), Kyoto University Hospital, Kyoto, Japan; 6 Department of Therapeutic Oncology, Kyoto University Hospital, Kyoto, Japan; University of Kansas School of Medicine, United States of America

## Abstract

**Background:**

Individuals with serrated polyps (SP) are at higher risk for synchronous colorectal advanced neoplasms (AN) and cancers. However, it remains unclear whether there is a unique involvement of the serrated pathway and/or the classical adenoma-carcinoma sequence in this setting.

**Methods:**

Colorectal ANs, which include tubular adenomas ≥10 mm, adenomas with villous histology, high-grade intraepithelial neoplasms, and cancers, were collected retrospectively. The groups included ANs with (AN+SP) or without (AN-only) coexisting SPs. Clinicopathological findings were compared between groups. *BRAF* and *KRAS* mutations in ANs and SPs, and methylation levels at long interspersed element-1 (LINE-1) in adjacent mucosa were determined by pyrosequencing.

**Results:**

Seventy-five ANs from 40 patients in the AN+SP group, and 179 ANs from 119 patients in the AN-only group were analyzed. There were no significant differences in clinicopathological findings between the two groups, except that intraepithelial neoplasia in the AN+SP group was more likely to be located in the right colon (*P* = 0.018). *BRAF* mutations were significantly more frequent in the AN+SP group (*P* = 0.003), while KRAS mutations showed no significant differences between groups (*P* = 0.142). The majority of high-grade intraepithelial neoplasms in both groups showed a contiguous component of conventional adenoma. Individuals with large and right-sided SPs had significantly more conventional adenomas compared to those without such SPs (*P* = 0.027 and *P* = 0.031, respectively). Adjacent mucosa from individuals with multiple and large SPs showed significantly lower methylation levels at LINE-1 compared to individuals without such associated SPs (*P* = 0.049 and *P* = 0.015, respectively).

**Conclusion:**

Our data suggest that both the adenoma-carcinoma sequence and the serrated pathway are operational in individuals with coexisting ANs and SPs. The reduced methylation levels at LINE-1 in the background mucosa suggest the possibility of an underlying ‘field defect’.

## Introduction

Colorectal cancer (CRC) evolves through multiple distinct pathways, including the classical adenoma-carcinoma sequence and the serrated pathway [1]. These pathways are defined based on molecular features and the pathology of the precursor lesions. The classical adenoma-carcinoma sequence originates in conventional adenomas (tubular adenomas and tubulovillous adenomas), whereas the serrated pathway develops in serrated polyps (SP).

SPs are characterized histologically by the serrated architecture of the crypt epithelium, and are grouped into three categories: hyperplastic polyps (HP), sessile serrated adenoma/polyps (SSA/P) and traditional serrated adenomas (TSA) [2], [3]. HPs are common in the distal colorectum, and have little or no malignant potential. SSA/Ps are typically located in the proximal colon, and are often larger than HPs. By contrast, TSAs are more common in the distal colon, and have a reddish and protuberant appearance [3], [4]. Importantly, SSA/Ps and TSAs are potential precursor lesions of CRC in the serrated pathway. The process of colorectal carcinogenesis via the serrated pathway is characterized by presence of mutations in the *BRAF* gene, the CpG island methylator phenotype, and microsatellite instability, which occurs as a consequence of methylation-induced, biallelic silencing of the *MLH1* gene. [1], [3], [5]

Some SPs are precancerous lesions *per se*, but they also serve as markers for the presence of coexisting adenomas and/or CRCs. It has been reported that HPs, most of which were located in the distal colon, can predict the presence of adenomas throughout the colon [6]. A meta-analysis revealed that patients with distal HPs have an intermediate risk for proximal colorectal neoplasia, however, they also mentioned that this increased risk disappeared if only high-quality studies on screening patients were considered [7]. More recently, those with large and/or proximal SPs have been reported to be at higher risk for synchronous advanced neoplasia (AN) and CRC [8]–[10]. These observations suggest that those who harboring SPs may carry an increased risk for CRC, and raise the importance of these individuals as a model of a “high risk colon”. Interestingly, recent study by Rosty et al. [11] reported that the majority of CRCs arising in serrated polyposis, a CRC predisposition syndrome characterized by the presence of multiple SPs, did not harbor molecular hallmarks of serrated pathway. Their data suggest the involvement of both the serrated pathway and the adenoma-carcinoma sequence in the carcinogenesis in serrated polyposis; however, it remains unclear whether there is a unique involvement of the serrated pathway and/or the classical adenoma-carcinoma sequence, or whether both pathways participate in the setting associated with sporadic SPs.

Genetic and epigenetic alterations can exist in the background colorectal mucosa of a “high risk colon” [12]. Indeed, hypermethylation of cancer-related genes have been reported in normal-appearing mucosa of patients with CRC [13]–[17] as well as SPs [18], [19]. Methylation of *Long Interspersed Element-1* (*LINE-1*) DNA sequences is a surrogate marker for global DNA methylation [20], [21]; furthermore, hypomethylation of *LINE-1* elements has been associated with chromosomal instability in CRC [22] as well as poor prognosis for CRC patients [23]. *LINE-1* hypomethylation has been found to be significantly associated in the case of synchronous CRC pairs from the same patient [24]. In addition, adjacent normal-appearing colonic mucosa from patients with multiple synchronous CRCs show significant hypomethylation compared with that from patients with a single lesion, or healthy volunteers [25]. Based on these observations, we hypothesized that *LINE-1* hypomethylation in colorectal mucosa may play an important role in establishing a ‘field defect’ in colorectal carcinogenesis.

In this study, we proposed to gain insight into this process by analyzing patients who had both colorectal ANs and SPs, as these provide as a model of high risk for CRC. The aims of this study were to first interrogate the clinicopathological and molecular characteristics of ANs that coexist with SPs, and second, to determine whether *LINE-1* hypomethylation in the background mucosa of patients with AN and SP constitutes a methylation ‘field defect’, which may be responsible for accelerated tumor progression in these patients.

## Materials and Methods

### Patients

Records of colonoscopy performed at the Kyoto University Hospital from January 2007 and December 2010 were reviewed retrospectively. Patients who underwent colonoscopy and subsequently received endoscopic or surgical resection for colorectal ANs were enrolled. An AN was defined by the presence of tubular adenomas ≥10 mm, adenomas with any villous histology, adenomas with high-grade intraepithelial neoplasia (HGIN), or invasive adenocarcinomas [8]–[10]. The AN+SP group included patients with colorectal ANs who had synchronous and/or metachronous SPs. The control group consisted of patients with colorectal AN alone without SPs (AN-only group). Patients with familial adenomatous polyposis, Lynch syndrome, serrated polyposis, inflammatory bowel disease, a history of chemotherapy and/or radiotherapy were excluded. Those patients who showed the presence of suspicious or possible SP by endoscopic examination were also excluded. Written informed consent was obtained from all patients, and the study protocol was approved by the Institutional Review Board of the Kyoto University Graduate School and Faculty of Medicine.

All endoscopic reports, endoscopic images and pathological reports of enrolled cases were reviewed by one of the authors (AY). Morphology of intraepithelial neoplasia was classified according to the Paris endoscopic classification of superficial neoplastic lesions [26], and designated as either protruded (0–1 p and 0–1 sp) or superficial (0–1 s, 0–2 a, 0–2 c, and mixed type). A pathological diagnosis of AN and SP was made based on WHO classification criteria [3]. Tubular and tubulovillous adenomas were designated as conventional adenomas in this study. All histological slides of SPs were reviewed by an expert gastrointestinal pathologist (SM). SPs were classified as HPs, SSA/Ps, or TSAs. SPs that did not convincingly fit into any of these categories were classified as a serrated lesion (SL). SPs larger than 10 mm were designated as a large SP. Clinicopathological information was obtained from medical charts, and assessed based on the International Union Against Cancer (UICC) TNM staging system [27]. Survival analyses were conducted on 123 patients who had invasive cancer. The median follow-up period of these patients was 32.6 months (range, 0.7–93.1).

### DNA extraction

Tissue samples of colorectal ANs, SPs, and adjacent mucosa were microdissected from 10 µM thick formalin-fixed paraffin-embedded sections. Genomic DNA was extracted either using the QIAamp DNA FFPE Tissue Kit (QIAGEN, Hilden, Germany), or for smaller lesions and adjacent mucosa, the QIAamp DNA Investigator Kit (QIAGEN), according to the manufacturer's instructions. For SPs, DNA was extracted only from endoscopically and surgically resected lesions, but not from biopsy specimens. Samples of adjacent mucosa were obtained from mucosa which appeared histologically normal, at least 30 mm away from the corresponding AN. In most situations, adjacent mucosa was collected from the proximal or distal end of the surgically resected specimen.

### Mutation analysis for *BRAF* and *KRAS* by pyrosequencing

DNA from ANs and SPs were subjected to PCR amplification of exon 15 of the *BRAF* gene and exon 2 of the *KRAS* gene. PCR amplification was performed in 25 µL reactions containing 12.5 µl of HotStarTaq Master Mix Kit (QIAGEN), 5 pmol each of forward and reverse primers, and 50 ng of template DNA. PCR conditions were as follows: initial Taq activation at 95°C for 15 minutes, 40 cycles of denaturation at 94°C for 30 seconds, annealing at 52°C for *BRAF* or 59°C for *KRAS* for 30 seconds, elongation at 72°C for 1 minute, and final extension at 72°C for 10 minutes. Mutations in the *BRAF* (codon 600) and *KRAS* (codon 12 and 13) genes were analyzed by pyrosequencing. Pyrosequencing was performed using the PyroMark MD system (QIAGEN) as described previously [28]. Direct sequencing was performed to confirm the presence of mutations in *BRAF* and *KRAS* using the BigDye Terminator V1.1 Cycle Sequencing Kit and genetic analyzer (ABI 3130, Applied Biosystems, Foster, CA, USA). Primer sequences are shown in Table 1.

**Table 1:** PCR primers used in this study.

***BRAF***	Forward	GAA GAC CTC ACA GTA AAA ATA G
	Reverse	Bio-ATA GCC TCA ATT CTT ACC ATC C
	Sequencing	AGG TGA TTT TGG TCT AGC TAC AG
***KRAS***	Forward	GGC CTG CTG AAA ATG ACT GA
	Reverse	Bio-TAG CTG TAT CGT CAA GGC ACT CT
	Sequencing	TTG TGG TAG TTG GAG CT
***LINE-1***	Forward	TTT TGA GTT AGG TGT GGG ATA TA
	Reverse	Bio-AAA ATC AAA AAA TTC CCT TTC
	Sequencing	AGT TAG GTG TGG GAT ATA GT

### DNA methylation analysis

DNA from adjacent mucosa was bisulfite-modified using the EZ Methylation Gold Kit (Zymo Research, Irvine, CA, USA) according to the manufacturer's instructions. Bisulfite-converted DNA was subjected to PCR amplification for *LINE-1* methylation analysis. PCR amplification was performed in 25 µL reactions containing 12.5 µL of HotStarTaq Master Mix Kit (QIAGEN), 6.25 pmol each of forward and reverse primers, and 2 µL of bisulfate-converted DNA. PCR conditions were as follows: initial Taq activation at 95°C for 15 minutes, 50 cycles of denaturation at 94°C for 30 seconds, annealing at 51°C for 45 seconds, elongation at 72°C for 45 seconds, and final extension at 72°C for 10 minutes. DNA methylation was analyzed quantitatively by pyrosequencing using the PyroMark MD system (QIAGEN) [28], and methylation levels of *LINE-1* elements was calculated as the mean percentage of the four CpG sites analyzed. Primer sequences are shown in Table 1.

### Statistical analysis

The Mann-Whitney U test was used to compare continuous variables. To analyze categorical data, the Fisher's exact test or the chi-square test was used. Cumulative survival curves were drawn by the Kaplan-Meier method and the differences between the curves were analyzed by the log-rank test. All *P*-values were two-sided and a *P*-value of <0.05 was considered significant. Since this is an exploratory research, adjustments for multiple comparisons were not considered. All analyses were carried out using the JMP 10 (SAS institute Inc., Cary, NC, USA).

## Results

### Patient characteristics

There were 40 patients in the AN+SP group, 119 patients in the AN-only group with a total of 75 and 179 ANs, respectively. Of these, tissue samples for DNA analysis were available for 75 ANs from the AN+SP group and 174 ANs from the AN-only group. There were 75 SPs in the patients from the AN+SP group, and tissue samples for DNA analysis were available from a total of 39 SPs. Patients' characteristics are shown in Table 2. There were significantly more males than females in the AN+SP group. There were no other significant differences between the two groups in terms of age, personal and family history of CRC.

**Table 2:** Patients' characteristics.

		AN+SP group (n = 40)	AN-only group (n = 119)	*P-value*
**Age**	Median (Range)	72 (38–87)	68 (41–90)	0.083
**Gender**	Male (%)	30 (75.0)	67 (56.3)	0.040
	Female (%)	10 (25.0)	52 (43.7)	
**Personal history of CRC**	Present (%)	3 (7.5)	5 (4.2)	0.416
	Absent (%)	37 (92.5)	114 (95.8)	
**Family history of CRC** a	Present	6 (18.8)	17 (17.2)	0.795
	Absent	26 (81.2)	82 (82.8)	

a Data were not available in 28 cases.

### Clinicopathological and molecular characteristics of SPs

Characteristics of SPs are summarized in Table 3. Fifty-six of 75 SPs were located on the left side, while the remaining 19 were on the right side. The median size of the SPs was 5 mm (range 1–20). Fifty-nine SPs were superficial, while the remaining 16 were of a protruded morphology. Tissue samples were obtained by biopsy from 28 SPs, by endoscopic resection from 13 SPs, and by surgical resection from 34 SPs. All surgically resected SPs were located in close proximity to cancer, hence resected simultaneously. Histologically, SPs were classified as 35 HPs, 17 SSA/Ps, and 9 TSAs. Fourteen SPs could not be classified, and were thus referred to as SL. Twenty of 39 (51.3%) of the SPs contained *BRAF* gene mutations and 5/39 (12.8%) contained mutations in the *KRAS* gene. Among informative lesions, 5/19 HPs, 7/10 SSA/Ps, 5/6 TSAs, and 3/4 SLs had *BRAF* mutations, whereas 2/19 HPs, 2/10 SSA/Ps, and 1/4 SLs had *KRAS* mutations. No TSAs harbored mutations in the *KRAS* gene. Mutations in the *BRAF* and *KRAS* genes were mutually exclusive in all SPs.

**Table 3:** Characteristics of serrated polyps (SP) (n = 75)^a^.

Size (mm)	Median (Range)	5 (1–20)
**Location**	Right (%)	19 (25.3)
	Left (%)	56 (74.7)
**Morphology**	Protruded (%)	16 (21.3)
	Superficial (%)	59 (78.7)
**Pathological diagnosis**	Hyperplastic polyp (%)	35 (46.7)
	Sessile serrated polyp/adenoma (%)	17 (22.7)
	Traditional serrated adenoma (%)	9 (12.0)
	Serrated lesion (%)	14 (18.7)
***BRAF***	Wild type (%)	19 (48.7)
	Mutant (%)	20 (51.3)
***KRAS***	Wild type (%)	34 (87.2)
	Mutant (%)	5 (12.8)

a DNA samples for mutation analyses were available for 39 SPs.

### Clinicopathological characteristics of colorectal ANs

**Figure 1:**
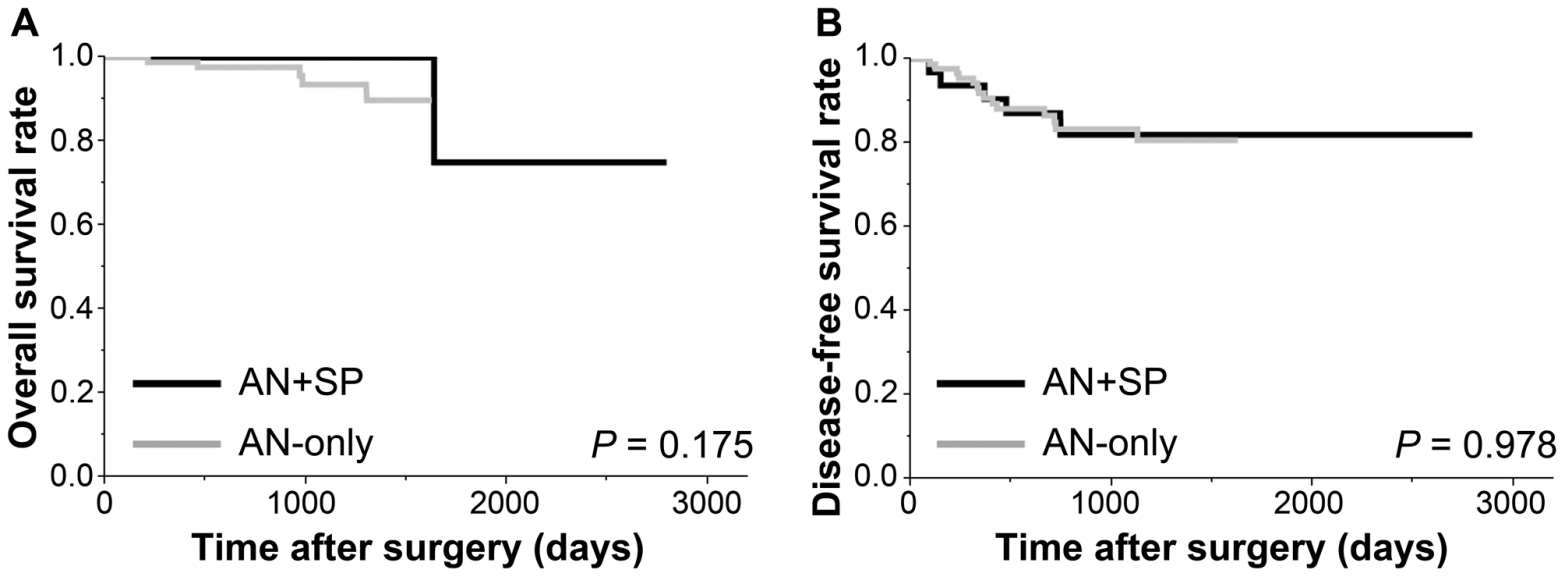
Survival analyses for patients with invasive cancer. Cumulative overall (A) and disease-free (B) survival rates showed no differences between AN+SP and AN-only groups.

Clinicopathological characteristics of colorectal ANs are shown in Table 4 and Table 5. Intraepithelial neoplasms in the AN+SP group were more likely in the proximal colon than in the AN-only group (Fisher's exact test, *P* = 0.018). Otherwise, there were no significant differences between these two groups in terms of morphology, size, or histology of intraepithelial neoplasms. No significant differences were seen between groups in the clinicopathological features of CRCs. Survival analyses for patients with CRCs revealed no differences in overall and disease-free survival rates between groups (Figure 1).

**Table 4:** Clinicopathological characteristics of colorectal intraepithelial neoplasms in AN+SP and AN-only groups.

		AN+SP group (n = 40)	AN-only group (n = 83)	*P-value*
**Location**	Right (%)	23 (57.5)	28 (33.7)	0.019
	Left (%)	17 (42.5)	55 (66.3)	
**Size (mm)**	Median (Range)	13 (8–40)	12 (5–105)	0.218
**Morphology**	Protruded (%)	21 (52.5)	48 (57.8)	0.698
	Superficial (%)	19 (47.5)	35 (42.2)	
**Histology**	Tubular adenoma (%)	20 (50.0)	39 (47.0)	0.820
	Tubulo-villous adenoma (%)	5 (12.5)	14 (16.9)	
	High-grade intraepithelial neoplasia (%)	15 (37.5)	30 (36.1)	

**Table 5:** Clinicopathological characteristics of colorectal cancers (CRC) in AN+SP and AN-only groups.

		AN+SP group (n = 35)	AN-only group (n = 96)	*P-value*
**Location**	Right (%)	4 (11.4)	19 (19.8)	0.312
	Left (%)	31 (88.6)	77 (80.2)	
**Size (mm)**	Median (Range)	40 (10–105)	35 (8–110)	0.706
**Grade** a	Well differentiated (%)	2 (5.7)	16 (16.7)	-
	Moderately differentiated (%)	32 (91.4)	77 (80.2)	
	Poorly differentiated (%)	1 (2.9)	3 (3.1)	
**T**	1 (%)	5 (14.3)	15 (15.6)	0.961
	2 (%)	10 (28.6)	23 (24.0)	
	3 (%)	14 (40.0)	41 (42.7)	
	4 (%)	6 (17.1)	17 (17.7)	
**N**	0 (%)	26 (74.3)	63 (66.3)	0.616
	1 (%)	6 (17.1)	24 (25.3)	
	2 (%)	3 (8.6)	8 (8.4)	
**M**	0 (%)	33 (94.3)	88 (91.7)	1.000
	1 (%)	2 (5.7)	8 (8.3)	
**Stage** b	1 (%)	13 (37.1)	31 (32.6)	0.848
	2 (%)	12 (34.3)	29 (30.5)	
	3 (%)	8 (22.9)	27 (28.4)	
	4 (%)	2 (5.7)	8 (8.4)	

a Statistical analysis was not conducted because of the small sample number.

b Lymph nodes metastasis status and stage could not be determined in one lesion.

### Mutational analysis of *BRAF* and *KRAS* in colorectal ANs

**Figure 2:**
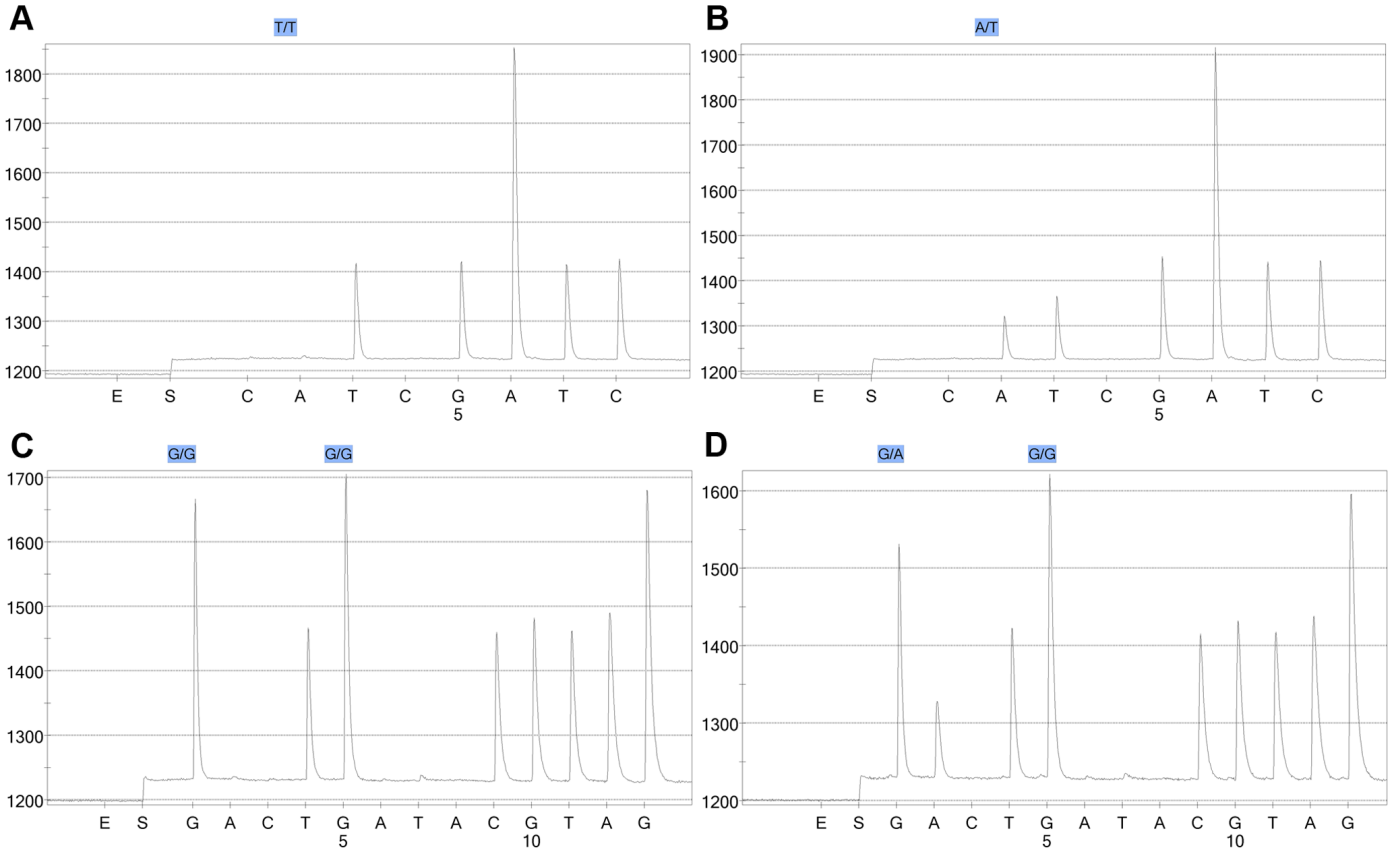
Representative results of the pyrosequencing examining *BRAF* and *KRAS* mutations in ANs. Pyrograms of the representative cases showing *BRAF* wild-type (A), *BRAF* mutation (B, T to A mutation at codon 600), *KRAS* wild-type (C), *KRAS* mutation (D, G to A mutation at codon 12).

Representative pyrograms for *BRAF* and *KRAS* mutation are shown in Figure 2, and the results of the mutation analyses are summarized in Table 6. There were more mutations in the *BRAF* gene (codon 600) in the AN+SP group (6/75 ANs) compared to in only 1/174 ANs in the AN-only group (Fisher's exact test, *P* = 0.003). Interestingly the only AN in the AN-only group with a *BRAF* mutation was a HGIN with a contiguous TSA component. There was no difference in the frequency of *KRAS* mutations (codon 12 and 13) between the two groups (*P* = 0.142). As was the case with SPs, mutations in the *BRAF* and *KRAS* genes were mutually exclusive, and 50/75 (66.7%) ANs in AN+SP group and 112/174 (64.4%) ANs in AN-only group had no detectable mutations in either the *BRAF* or *KRAS* gene.

**Table 6:** Mutational status of the *BRAF* and *KRAS* genes in advanced neoplasms (AN).

		AN+SP group (n = 75)	AN-only group (n = 174)	*P-value*
***BRAF***	Wild type (%)	69 (92.0)	173 (99.4)	0.003
	Mutant (%)	6 (8.0)	1 (0.6)	
***KRAS***	Wild type (%)	56 (74.7)	113 (64.9)	0.142
	Mutant (%)	19 (25.3)	61 (35.1)	
***BRAF*** ** and ** ***KRAS*** a	Wild type for both genes (%)	50 (66.7)	112 (64.4)	0.773
	Mutant for either *BRAF* or *KRAS* (%)	25 (33.3)	62 (35.6)	

a *BRAF* and *KRAS* mutations were mutually exclusive.

### Relationship between the existence of SP and the adenoma-carcinoma sequence

**Figure 3:**
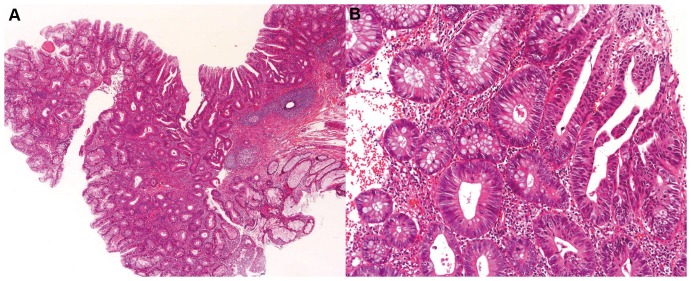
A representative case of high-grade intraepithelial neoplasia (HGIN) with contiguous conventional adenoma. Histological images (H&E) of an AN which shows contiguous parts of a tubular adenoma and a HGIN in the same lesion. (A: low magnification, B: high magnification).

HGINs frequently have a contiguous component of low-grade neoplasia, which is thought to be one of the precursor steps in the evolution of high-grade neoplasia. We examined the HGINs for the histology of a contiguous low-grade neoplastic component. Among 45 HGINs, 14/15 and 27/30 in the AN+SP and AN-only groups, respectively, showed a contiguous component of conventional adenoma (Figure 3). There was one HGIN in the AN-only group which showed a contiguous component of TSA as mentioned above. No significant differences were observed between groups (Table 7).

**Table 7:** Presence of contiguous components of conventional adenomas and serrated polyps (SP) in high-grade intraepithelial neoplasms (HGIN).

		AN+SP group (n = 15)	AN-only group (n = 30)	*P-value*
**Component of conventional adenoma**	Present (%)	14 (93.3)	27 (90.0)	1.000
	Absent (%)	1 (6.7)	3 (10.0)	
**Component of SP**	Present (%)	0 (0.0)	1 (3.3)	1.000
	Absent (%)	15 (100.0)	29 (96.7)	

**Figure 4:**
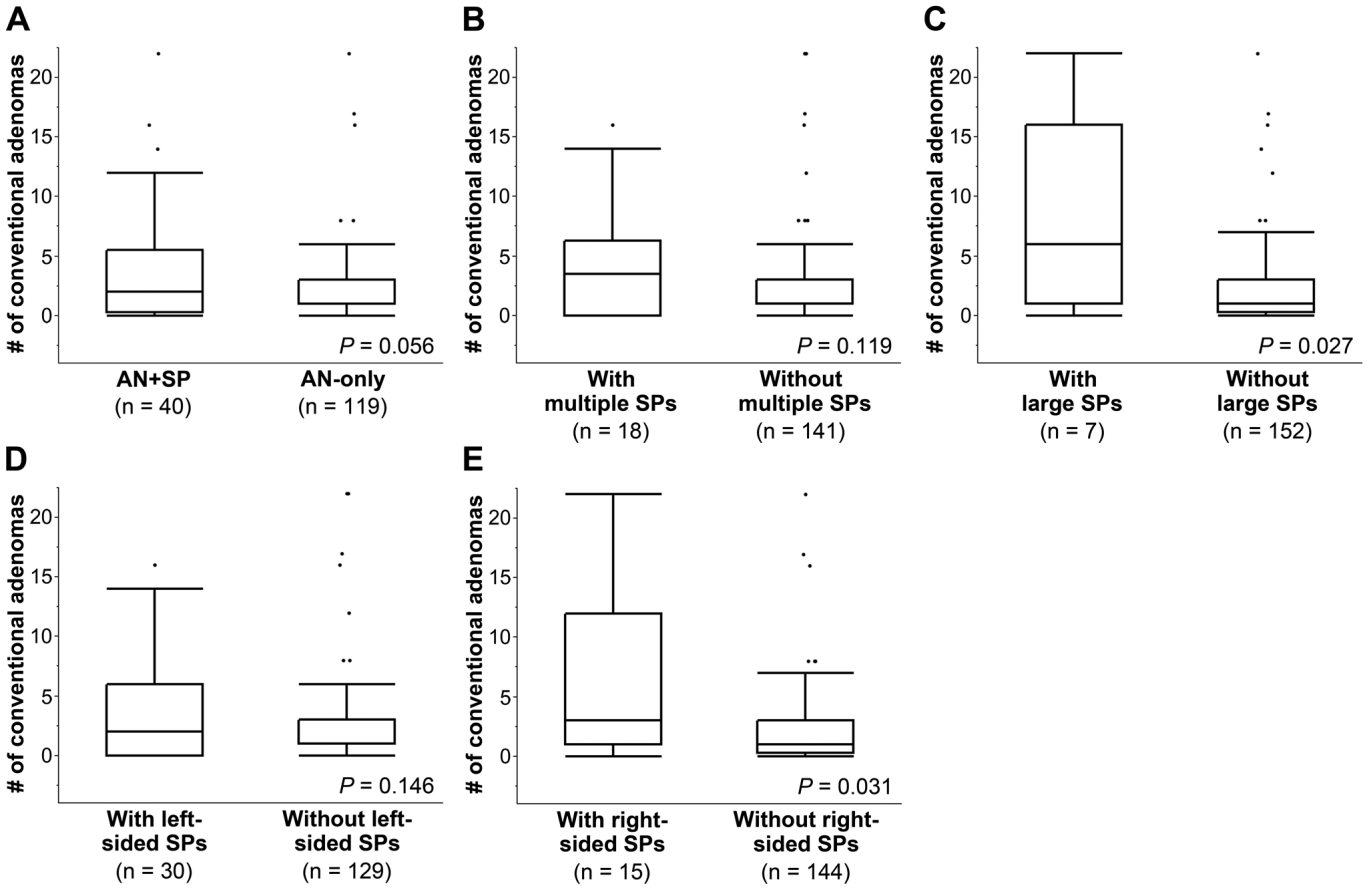
Multiplicity of conventional adenomas in patients with and without SPs. Number of conventional adenomas in each individual is compared between AN+SP and AN-only groups (A), individuals with and without multiple (B), large (C), left-sided (D), and right-sided (E) SPs. Statistical analyses were performed using the Mann-Whitney U test.

**Figure 5:**
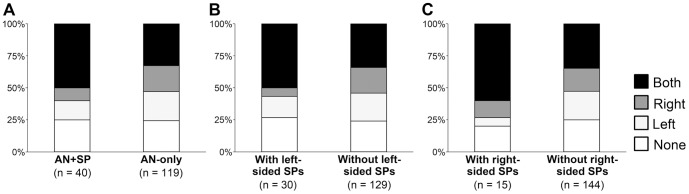
Location of conventional adenomas. Presence and location of conventional adenomas is compared between AN+SP and AN-only groups (A), individuals with and without left-sided SPs (B) and individuals with and without right-sided SPs (C). Statistical analyses were not conducted because of small sample sizes.

Next, we compared the number of conventional adenomas in relation to SPs in each individual to further explore the associations between SPs and conventional adenomas. As shown in Figure 4A, individuals in the AN+SP group tended to have more conventional adenomas than those in the AN-only group (Mann-Whitney U test, *P* = 0.056). Individuals with large and right-sided SPs harbored significantly more conventional adenomas than those without such SPs (Figure 4C,E; Mann-Whitney U test, *P* = 0.027 and *P* = 0.031, respectively). More than half of the individuals with SPs had conventional adenomas in both sides of the colorectum regardless of the location of SPs (Figure 5).

### LINE-1 hypomethylation in mucosa adjacent to ANs

**Figure 6:**
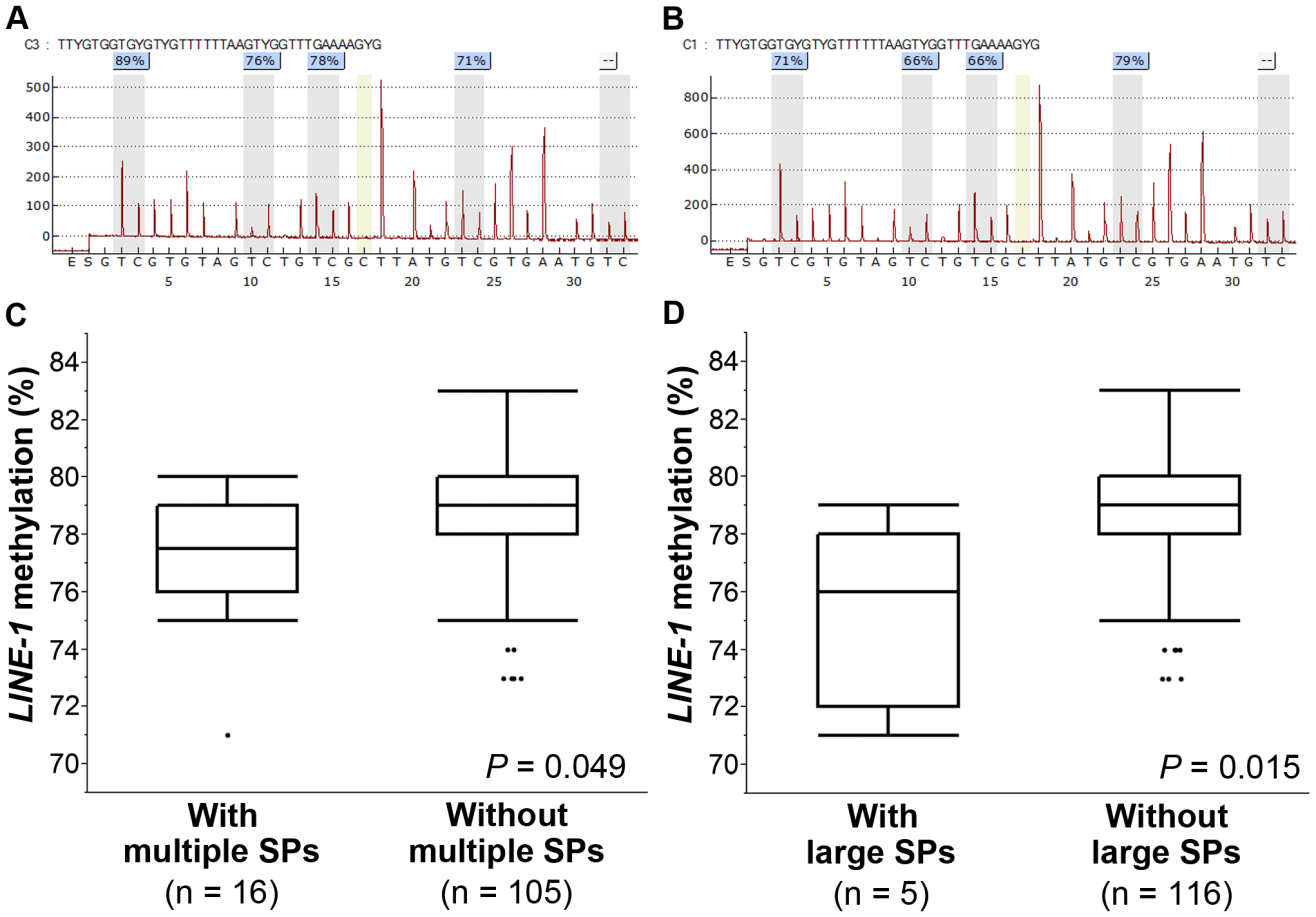
*LINE-1* methylation levels in adjacent mucosa. Representative results of bisulfite pyrosequencing showing the levels of *LINE-1* methylation in adjacent mucosa which was calculated as the mean percentage of the four CpG sites (A and B). Individuals with multiple (C) and large (D) SPs showed significantly lower *LINE-1* methylation levels compared to individuals without such SPs. Statistical analyses were performed using the Mann-Whitney U test.

To explore if there were alterations in the background mucosa that could contribute to carcinogenesis in the AN+SP group individuals, we examined DNA methylation of *LINE-1* elements in histologically normal mucosa adjacent to the ANs. Tissue samples of adjacent normal-appearing mucosa were available from 35 and 86 patients in the AN+SP and AN-only groups, respectively. Figure 6A and 6B show representative results of bisulfite pyrosequencing examining the methylation levels of *LINE-1*. There were no significant differences in *LINE-1* methylation levels between AN+SP and AN-only groups (data not shown). However, those patients who had multiple SPs showed significantly lower *LINE-1* methylation levels than individuals with one or fewer SPs (Figure 6C; Mann-Whitney U test, *P* = 0.049). Moreover, individuals with large SPs had significantly lower *LINE-1* methylation levels in their background mucosa than those without large SPs (Figure 6D; Mann-Whitney U test, *P* = 0.015).

## Discussion

In the current study, we investigated the clinicopathological and molecular characteristics of colorectal ANs in the context of the presence or absence of coexisting SPs. The fact that our clinicopathological findings showed similarities between ANs alone or in the presence of SPs may be a reflection of the common characteristics of ANs in both groups, rather than the possibility that this disease is derived from distinct pathways.

We found that mutations in the *BRAF* gene were significantly more frequent in ANs with coexisting SPs than in ANs without coexisting SPs. Since *BRAF* gene mutations are regarded as a hallmark of carcinogenesis via the serrated pathway (1,3,5), these results are consistent with the concept that individuals with SPs perhaps evolve via the serrated pathway, and eventually some of these proceed and progress to cancer. In addition to mutations of the *BRAF* gene, mutations in *KRAS* have been also implicated in the serrated pathway [1], [3], [5]. In the present study, 66.7% and 64.4% of ANs in the AN+SP and AN-only group, respectively, showed no mutations in either *BRAF* or *KRAS*. Because mutations in *BRAF* and *KRAS* are thought to play a key role in the early development of SPs [1], [3], majority of ANs which did not harbor a mutation in *BRAF* or *KRAS* were more likely derived from lesions other than a SP.

To further confirm this hypothesis, we histologically examined HGINs, which represent the transition lesion in malignant transformation. We found that 14/15 HGINs in AN+SP group possessed a contiguous component of conventional adenoma, whereas these same lesions lacked a SP component. Similarly, 27/30 HGINs had a contiguous component of conventional adenoma, while only 1/30 lesions showed a component of SP in the AN-only group. These observations directly indicate that the majority of ANs both with and without coexisting SPs develop through the adenoma-carcinoma sequence rather than the serrated pathway. In serrated polyposis, Rosty et al. [11] reported 47.3% of CRCs had *BRAF* mutation while another 47.3% were *BRAF*/*KRAS* wild type, and 4/13 CRCs with contiguous residual polyp harbored conventional adenomas. Our data are in line with the observations made by Rosty and colleagues, and highlight the involvement of both the adenoma-carcinoma sequence and the serrated pathway in the settings of colorectum harboring sporadic SPs as well as serrated polyposis. It is worth noting that far more ANs in our AN+SP group seemed to be associated with the adenoma-carcinoma sequence. This may reflect the heterogeneity of SPs and inclusion of non-risky SPs to our AN+SP group. In addition, we also showed that individuals with ANs and coexisting SPs tended to possess more conventional adenomas than those who had ANs without coexisting SPs. In particular, those AN patients with large and right-sided SPs had significantly more conventional adenomas compared to AN patients without such SPs. These data are consistent with previous reports [9], [29], and support the hypothesis that individuals with SPs have an accelerated neoplastic progression in the adenoma-carcinoma sequence. Taken together, results of ours and others suggest that both the classical adenoma-carcinoma sequence and the serrated pathway may be operational in individuals bearing ANs and SPs.

Although the results of our study suggest that dual carcinogenesis pathways are present in individuals with AN and SP, the mechanism(s) underlying their neoplastic progression remain unclear. Previous studies have demonstrated the association between the presence of large SPs and synchronous ANs in the same individuals. Interestingly, coexisting ANs and SPs were not necessarily in the same side of the colon. [8], [10] In the present study, we demonstrated that more than half of individuals with co-existing ANs and SPs had conventional adenomas in both sides of colorectum. Based on our observations and those of others, it is conceivable that the molecular alteration(s) which potentially affect colorectal mucosa extensively to promote carcinogenesis may be present. To date, several reports have demonstrated ‘field defects’ of colorectal mucosa caused by abnormal DNA methylation [13]–[17]. In the current study, we observed that the adjacent mucosa in individuals with ANs and coexisting multiple and large SPs had significantly lower levels of *LINE-1* methylation compared to the adjacent mucosa in those who had ANs without such associated SPs. Since hypomethylation of *LINE-1* can lead to activation of proto-oncogenes and are associated with chromosomal instability in CRC [22], lower methylation levels of *LINE-1* seen in adjacent mucosa may play a role in the process of carcinogenesis in these individuals with multiple or large SPs. To validate this hypothesis, prospective studies with greater numbers of patients are needed.

With regards to potential limitations of our study, as a retrospective study, we had no control over whether all of the SPs were collected from all patients. SPs are frequently found during colonoscopy but not all were followed up with histological examination since the decision to obtain a biopsy was at the physician's discretion. This could have biased our results, since only patients with histologically proven SPs were enrolled in this study. Conversely, patients with SPs could have been missed and placed into the AN-only group. To minimize this potential problem for our AN-only group, we reviewed all colonoscopic images to exclude patients with possible SPs. A second potential limitation was that more than one third of the SPs were not resected but tissue was collected by biopsies. Histological classification of SPs is often difficult in the biopsy specimen alone. In this study, 14 SPs could not be histologically classified into a specific category, and thus were placed in a separate group, called SL. A total of 14 SPs were classified as SLs, and 10/14 SLs had only a biopsy for analysis. In addition, we were not able to obtain DNA for mutation analyses from SPs which were not resected, and had only a biopsy specimen.

In conclusion, our data highlight that both the adenoma-carcinoma sequence and the serrated pathway appear to be operative in individuals with both ANs and SPs. The reduced levels of *LINE-1* methylation in the background mucosa suggest the possibility of an underlying ‘field defect’ in patients with multiple or large SPs; however, further study is required to determine the significance of this phenomenon.

## Supporting Information

Information S1 **Clinicopathological and mutation data for advanced neoplasms.**
(XLSX)

Information S2 **Clinicopathological and mutation data for serrated polyps.**
(XLSX)

Information S3 **Levels of LINE-1 methylation in adjacent mucosa.**
(XLSX)

## References

[1] Jass JR (2007) Classification of colorectal cancer based on correlation of clinical, morphological and molecular features. Histopathology 50: 113–130. 17204026 10.1111/j.1365-2559.2006.02549.x

[2] Torlakovic E, Skovlund E, Snover DC, Torlakovic G, Nesland JM (2003) Morphologic reappraisal of serrated colorectal polyps. Am J Surg Pathol 27: 65–81. 12502929 10.1097/00000478-200301000-00008

[3] Snover DC, Ahnen DJ, Burt RW, Odze RD (2010) Serrated polyps of the colon and rectum and serrated polyposis. In: Bozman FT, Carneiro F, Hruban RH, Theise ND, editors. WHO classification of tumours of the digestive disease, fourth edition. Lyon: IARC Press. pp. 160–165.

[4] Yamada A, Notohara K, Aoyama I, Miyoshi M, Miyamoto S, et al (2011) Endoscopic features of sessile serrated adenoma and other serrated colorectal polyps. Hepatogastroenterology 58: 45–51. 21510285

[5] Rex DK, Ahnen DJ, Baron JA, Batts KP, Burke CA, et al (2012) Serrated lesions of the colorectum: review and recommendations from an expert panel. Am J Gastroenterol 107: 1315–1329. 22710576 10.1038/ajg.2012.161 PMC3629844

[6] Jass JR, Young PJ, Robinson EM (1992) Predictors of presence, multiplicity, size and dysplasia of colorectal adenomas. A necropsy study in New Zealand. Gut 33: 1508–1514. 1452076 10.1136/gut.33.11.1508 PMC1379537

[7] Lin OS, Gerson LB, Soon MS, Schembre DB, Kozarek RA (2005) Risk of proximal colon neoplasia with distal hyperplastic polyps: a meta-analysis. Arch Intern Med 165: 382–390. 15738366 10.1001/archinte.165.4.382

[8] Li D, Jin C, McCulloch C, Kakar S, Berger BM, et al (2009) Association of large serrated polyps with synchronous advanced colorectal neoplasia. Am J Gastroenterol 104: 695–702. 19223889 10.1038/ajg.2008.166

[9] Schreiner MA, Weiss DG, Lieberman DA (2010) Proximal and large hyperplastic and nondysplastic serrated polyps detected by colonoscopy are associated with neoplasia. Gastroenterology 139: 1497–1502. 20633561 10.1053/j.gastro.2010.06.074

[10] Hiraoka S, Kato J, Fujiki S, Kaji E, Morikawa T, et al (2010) The presence of large serrated polyps increases risk for colorectal cancer. Gastroenterology 139: 1503–1510. 20643134 10.1053/j.gastro.2010.07.011

[11] Rosty C, Walsh MD, Walters RJ, Clendenning M, Pearson SA, et al (2013) Multiplicity and molecular heterogeneity of colorectal carcinomas in individuals with serrated polyposis. Am J Surg Pathol 37: 434–442. 23211288 10.1097/PAS.0b013e318270f748 PMC3567207

[12] Ushijima T (2007) Epigenetic field for cancerization. J Biochem Mol Biol 40: 142–150. 17394762 10.5483/bmbrep.2007.40.2.142

[13] Nakagawa H, Nuovo GJ, Zervos EE, Martin EW Jr, Salovaara R, et al (2001) Age-related hypermethylation of the 5′ region of MLH1 in normal colonic mucosa is associated with microsatellite-unstable colorectal cancer development. Cancer Res 61: 6991–6995. 11585722

[14] Shen L, Kondo Y, Rosner GL, Xiao L, Hernandez NS, et al (2005) MGMT promoter methylation and field defect in sporadic colorectal cancer. J Natl Cancer Inst 97: 1330–1338. 16174854 10.1093/jnci/dji275

[15] Kawakami K, Ruszkiewicz A, Bennett G, Moore J, Grieu F, et al (2006) DNA hypermethylation in the normal colonic mucosa of patients with colorectal cancer. Br J Cancer 94: 593–598. 16421593 10.1038/sj.bjc.6602940 PMC2361181

[16] Ahlquist T, Lind GE, Costa VL, Meling GI, Vatn M, et al (2008) Gene methylation profiles of normal mucosa, and benign and malignant colorectal tumors identify early onset markers. Mol Cancer 7: 94. 19117505 10.1186/1476-4598-7-94 PMC2639620

[17] Silviera ML, Smith BP, Powell J, Sapienza C (2012) Epigenetic differences in normal colon mucosa of cancer patients suggest altered dietary metabolic pathways. Cancer Prev Res 5: 374–384. 10.1158/1940-6207.CAPR-11-0336 PMC3294197 22300984

[18] Minoo P, Baker K, Goswami R, Chong G, Foulkes WD, et al (2006) Extensive DNA methylation in normal colorectal mucosa in hyperplastic polyposis. Gut 55: 1467–1474. 16469793 10.1136/gut.2005.082859 PMC1856423

[19] Worthley DL, Whitehall VL, Buttenshaw RL, Irahara N, Greco SA, et al (2010) DNA methylation within the normal colorectal mucosa is associated with pathway-specific predisposition to cancer. Oncogene 29: 1653–1662. 19966864 10.1038/onc.2009.449

[20] Yang AS, Estécio MR, Doshi K, Kondo Y, Tajara EH, et al (2004) A simple method for estimating global DNA methylation using bisulfite PCR of repetitive DNA elements. Nucleic Acids Res 32: e38. 14973332 10.1093/nar/gnh032 PMC373427

[21] Weisenberger DJ, Campan M, Long TI, Kim M, Woods C, et al (2005) Analysis of repetitive element DNA methylation by MethyLight. Nucleic Acids Res 33: 6823–6836. 16326863 10.1093/nar/gki987 PMC1301596

[22] Matsuzaki K, Deng G, Tanaka H, Kakar S, Miura S, et al (2005) The relationship between global methylation level, loss of heterozygosity, and microsatellite instability in sporadic colorectal cancer. Clin Cancer Res 11: 8564–8569. 16361538 10.1158/1078-0432.CCR-05-0859

[23] Ogino S, Nosho K, Kirkner GJ, Kawasaki T, Chan AT, et al (2008) A cohort study of tumoral LINE-1 hypomethylation and prognosis in colon cancer. J Natl Cancer Inst 100: 1734–1738. 19033568 10.1093/jnci/djn359 PMC2639290

[24] Nosho K, Kure S, Irahara N, Shima K, Baba Y, et al (2009) A prospective cohort study shows unique epigenetic, genetic, and prognostic features of synchronous colorectal cancers. Gastroenterology 137: 1609–1620. 19686742 10.1053/j.gastro.2009.08.002 PMC2859181

[25] Kamiyama H, Suzuki K, Maeda T, Koizumi K, Miyaki Y, et al (2012) DNA demethylation in normal colon tissue predicts predisposition to multiple cancers. Oncogene 31: 5029–5037. 22310288 10.1038/onc.2011.652 PMC4013778

[26] Endoscopic Classification Review Group (2005) Update on the Paris classification of superficial neoplastic lesions in the digestive tract. Endoscopy 37: 570–578. 15933932 10.1055/s-2005-861352

[27] Sobin LH, Gospodarowicz MK, Wittekind C (2009) TNM classification of malignant tumours, seventh edition. Oxford: Wiley-Blackwell.

[28] Goel A, Xicola RM, Nguyen TP, Doyle BJ, Sohn VR, et al (2010) Aberrant DNA methylation in hereditary nonpolyposis colorectal cancer without mismatch repair deficiency. Gastroenterology 138: 1854–1862. 20102720 10.1053/j.gastro.2010.01.035 PMC2859993

[29] Vu HT, Lopez R, Bennett A, Burke CA (2011) Individuals with sessile serrated polyps express an aggressive colorectal phenotype. Dis Colon Rectum 54: 1216–1223. 21904135 10.1097/DCR.0b013e318228f8a9

